# Prevalence and risk factors of Q fever (*Coxiella burnetii*) in cattle on farms of Limpopo province, South Africa

**DOI:** 10.3389/fvets.2023.1101988

**Published:** 2023-04-26

**Authors:** Vhahangwele Sadiki, Nomakorinte Gcebe, Maruping L. Mangena, Yusuf B. Ngoshe, Abiodun A. Adesiyun

**Affiliations:** ^1^Department of Production Animal Studies, Faculty of Veterinary Science, University of Pretoria, Pretoria, South Africa; ^2^Agricultural Research Council–Bacteriology and Zoonotic Diseases Diagnostic Laboratory, Onderstepoort Veterinary Research, Pretoria, South Africa; ^3^Agricultural Research Council–Transboundary Animal Diseases Programme, Onderstepoort Veterinary Research, Onderstepoort, South Africa; ^4^Faculty of Medical Sciences, School of Veterinary Medicine, University of the West Indies, St. Augustine, Trinidad and Tobago

**Keywords:** IS1111, PCR, South Africa, cattle, Q fever, *Coxiella burnetii*, ELISA

## Abstract

Q fever in animals and humans and its economic and public health significance has been widely reported worldwide but in South Africa. There are few studies on the prevalence of this zoonosis and its associated risk factors in South African livestock. Therefore, a cross-sectional study was conducted to determine the seroprevalence, molecular prevalence, and risk factors associated with *C. burnetii* in cattle on farms in South Africa’s Limpopo province. Out of 383 cattle tested for antibodies, the overall seroprevalence was 24.28%. Herd size of >150 (OR: 9.88; 95%CI: 3.92–24.89; *p <* 0.01) remained associated with *C. burnetii* seropositivity in cattle. For PCR detection, targeting IS*1111* fragment, cattle with no abortion history (OR: 0.37; 95%CI: 0.18–0.77; *p* < 0.01) and herd size of >150 (OR: 3.52; 95%CI: 1.34–9.24; *p* < 0.01) remained associated with *C. burnetii* positivity. The molecular prevalence in sheath scrapings and vaginal swabs by IS*1111* PCR was 15.67%. Cohen’s kappa agreement test revealed a fair agreement between the PCR and ELISA results (*k* = 0.40). Sequence analysis revealed that the amplicons had similarities to the *C. burnetii* transposase gene fragment, confirming the presence of the pathogen. The higher seroprevalence than molecular prevalence indicated a past *C. burnetii* infection, no bacterial shedding through vaginal mucus in cows, or preputial discharge in bulls. Similarly, the detection of *C. burnetii* by PCR in the absence of antibodies could be partly explained by recent infections in which antibodies have not yet been produced against the bacteria, or the level of these antibodies was below the detectability threshold. The presence of the pathogen in cattle and the evidence of exposure, as shown by both PCR and ELISA suggests an active circulation of the pathogen. This study demonstrated that *C. burnetii* is widespread in the study area and that a herd size of >150 is associated with *C. burnetii* seroprevalence and molecular prevalence.

## 1. Introduction

*Coxiella burnetii* is the etiology of Q fever or coxiellosis in animals and humans. The bacterium is Gram-negative coccobacillus which is obligately intracellular (1, 2). The bacterium belongs to the Gama-subdivision of Proteobacteria within the Legionellales order and family Coxiellaceae, according to 16S rRNA sequence analysis (1). It has been observed that the bacterium can survive in dairy, meat products, aborted foetuses, manure, clothes, and animal feed for a long period (3).

*Coxiella burnetii* infects a wide range of hosts, including humans, cattle, sheep, goats, and wild animals such as deer, buffaloes, squirrels, and rabbits (4). The main reservoirs for the bacterium are mostly cattle, sheep, and goats; ticks and rodents are considered the natural reservoirs (5, 6). *Coxiella burnetii* is shed in milk and vaginal secretions by goats and cows and in large numbers in faeces by sheep (7). *Coxiella burnetii* infection in domestic ruminants is mainly asymptomatic, and as such, the infection may remain unnoticed (3). The disease in these animals may result in huge economic losses as the disease causes reproductive disorders such as late abortions, weak or dead offspring in sheep and goats as well as infertility, abortion, metritis, and mastitis, in cattle which occur during later stages of infection (8, 9).

The disease has been reported worldwide except in New Zealand, and there are a few reports of *C. burnetii* infection in South African livestock (10, 11). Q fever seroprevalence in cattle has been reported in Zimbabwe (12), Kenya (13), Algeria (14) and in Nigeria (15). In the Netherlands the economic costs of the Q fever outbreak (2007–2010) in about 4,000 human cases acquired from infected dairy goat farms were estimated to be ~0.307 billion EUR (16). Human-to-human spread is rare, transmission to humans is mainly from contact with livestock or domestic animals, including consumption of raw milk, making the disease of public health significance (17, 18).

*Coxiella burnetii* seroprevalence in cattle has been reported in South Africa’s former Transvaal province, now, Gauteng province, where 7.80% of cattle were seropositive for the pathogen (19). Also, there has been a report on *C. burnetii* infections where 38.90% of cattle in Bushbuckridge municipality, Mpumalanga province, were positive (20). A recent abattoir-based study by Mangena et al. (10) in the Gauteng province of South Africa reported higher Q fever seroprevalence in cattle than in sheep and pigs. Q fever is of economical and occupational importance, and there is currently no data on seroprevalence and associated risk factors for transmission in the Limpopo Province of South Africa. There is limited data on the molecular prevalence of *C. burnetii* in cattle from South Africa. As such, we investigated the seroprevalence, molecular prevalence, associated risk factors, and the agreement of molecular and serological diagnostic of *C. burnetii* in cattle on farms in Limpopo province, South Africa.

## 2. Materials and methods

### 2.1. Ethical statement

Permission to conduct the study was obtained from the Animal Ethics Committee and the Research Committee of the Faculty of Veterinary Science (University of Pretoria). In addition, the Section 20 Ethical Approval Committee Certificate was granted for the study by the Department of Agriculture, Forestry, and Fisheries (DAFF). All the samples were collected with the assistance of the state animal health technicians. All the experiments were conducted under biosafety level III conditions at the Agricultural research council – Onderstepoort veterinary research.

### 2.2. Study area

**Figure 1:**
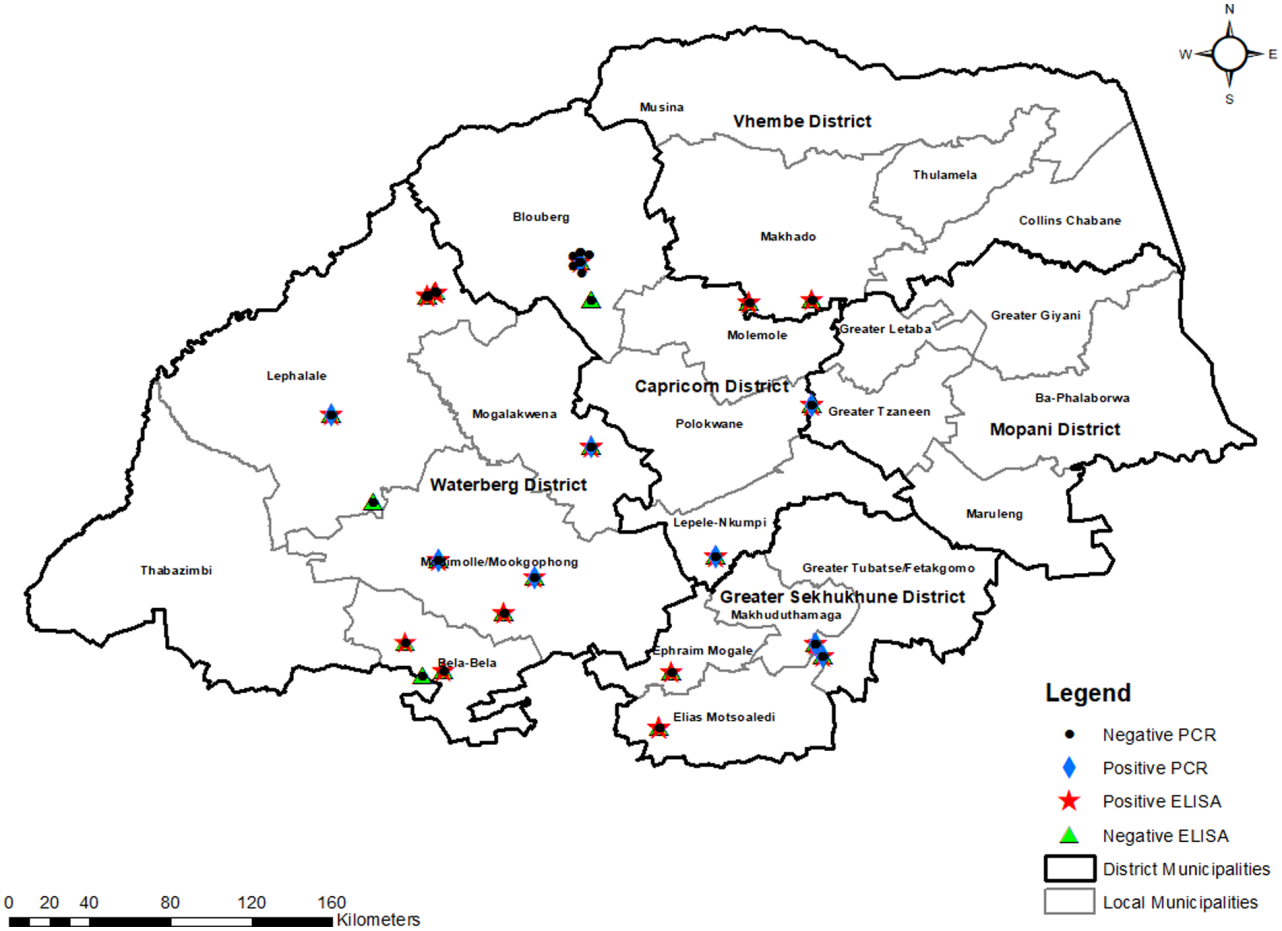
ELISA and PCR-based prevalence in the selected districts and local municipalities of Limpopo province.

This cross-sectional study was conducted in Limpopo province, South Africa’s northernmost, with a total area of 125,755 km^2^ and five district municipalities. Limpopo province is one of South Africa’s warmest regions, with an average daily high temperature of 26°C. For several month, temperatures are consistently above 25°C, sometimes reaching 29°C. The sampled district municipalities are Capricorn which is a stopover between Gauteng province and the northern areas of the country and between the north-western areas and the Kruger National Park and has a total area of 21,705 km^2^. Sekhukhune district lies in south-eastern part of the province with a total area of 13,528 km^2^. Lastly, Waterberg district is bordered to the north by Capricorn district municipality and to the south by Sekhukhune district municipality in the east. The Waterberg district has a total area of 44,913 km^2^.^1^ These district municipalities represent 50% of the districts in the province as other areas are foot and mouth disease (FMD) restricted areas. The study was conducted in the local municipalities that fall under each district municipality, namely Blouberg, Molemole, Polokwane, Lepelle Nkumpi (Capricorn), Lephalale, Bela-Bela, Mogalakwena and Modimolle (Waterberg) and Greater Tubatse, Ephraim Mogale, Elias Motsoaledi (Sekhukhune) (see text footnote 1; Figure 1).

### 2.3. Sample size and study population

The sample size for the study was calculated using the formula by Thrusfield (21) using an expected prevalence ( $P_{\exp})$ of 50% because no information on the prevalence of *Coxiella burnetii* in the study population was available. The desired precision (d) was set to 5%.

$$n_{0}=\frac{\left\{1.96^{2}\times P_{\exp}\times \left(1-P_{\exp}\right)\right\}}{d^{2}}$$

$$n_{0}=\frac{3.84\times 0.5\times 0.5}{0.0025}=\frac{0.96}{0.0025}=384$$

$$n_{0}=384\;\mathrm{Cattle}.$$

In this study, convenience sampling was used because only those farmers willing to participate in the research were included. Only adult and healthy cattle (bulls and cows) on the farms were included in the survey. A minimum of 128 cattle were to be sampled from each of the three districts in Limpopo province. A vast majority of the farms were communal in operation, but commercial farms were available and included in the study. A list of cattle farms, approximate cattle populations, and locations (GPS coordinates) or districts, and contact numbers of the owners were obtained from the Veterinary Division in the Limpopo province. Samples were collected from 22 cattle farms. The samples from each district were distributed according to the following criteria:

- Farms with <5 cattle: all animals were sampled.
- Farms with 5–20 cattle: a maximum of 8 were sampled.
- Farms with 21–30 cattle: a maximum of 12 were sampled.
- Farms with 31–50 cattle: a maximum of 18 were sampled.
- Farms with >50 cattle: maximum of 20 were sampled.

Samples were collected from nine farms with 1–50 cattle and four farms with 51–100 cattle. We also collected samples from eight farms with 101–150 cattle and only one farm with more than 150 cattle. A total of 383 cattle were sampled from all the local municipalities of the three district municipalities except two local municipalities that were excluded due to financial constraints.

### 2.4. Questionnaire survey

A consent form was issued to the farm owner or the attendant after explaining the study’s objectives and background on Q fever in Sepedi/English language to solicit their participation in the study. Prior to this sampling, the farmers and farm workers were unaware of Q fever. Also, a structured questionnaire survey was administered to the farmers in their local language (Sepedi) and in English to those that speak and understand the English language to collect demographic data and potential risk factors for exposure of animals and humans to *C. burnetii*. The information obtained from the questionnaire included the location of the farm or a dip tank in the province using GPS coordinates. Factors tested include district municipality, type of farm (commercial, communal), herd size (cattle number), sex, and tick infestation or presence of rodents in the farms, abortion history, infertility history, weak offspring history and if manure is used.

### 2.5. Sample collection

Samples were collected only from healthy adult cattle (bulls and cows) since no consent was given to sample calves or sick cattle. Blood samples were collected from the selected cattle from the coccygeal vein into tubes. Blood samples (10 ml) were collected using BD-Vacutainer^®^ SST™ II Advance 10 ml serum collection tubes. Sera were harvested by centrifuging the clotted blood in collection tubes at 1,000*g* for 10 min. The harvested sera were stored at −20°C until analysis. Vaginal swabs were collected from cows, including heifers using dry swabs dipped in 10 ml phosphate-buffered saline (PBS) (0.01 M, pH 7.4) and stored in sterile plastic bags. To collect sheath scrapings from bulls, scraping and simultaneous aspiration were performed with Uterine Infusion Pipettes (Kyron Laboratories, Johannesburg, South Africa) connected to a sterile disposable 10 ml hypodermic syringe with a silicon-rubber tube. Bulls were held in a sturdy crush with a neck clamp during sheath collection. Additional restraints included tying one back leg or using a Rau animal Immobilizer placed in the rectum to deliver low-level electrical stimulation. The samples were then transferred to plastic tubes containing 10 ml of PBS (0.01 M, pH 7.4).

### 2.6. Serological testing

Indirect enzyme-linked immunosorbent assay (iELISA) was used to screen serum samples for Q fever because of its high sensitivity and specificity (22). The IDEXX Q-FEVER 2/strip ELISA kit (IDDEX Laboratories, Liebelfld-Bern, Switzerland) was used to detect IgG antibodies to *C. burnetii* infection following the manufacturer’s instructions. Before use, all reagents (IDEXX Q Fever 2/strip antibody test kit reagents, as well as the frozen serum samples) were allowed to reach room temperature. Before use, the wash concentrate (10×) was diluted 1/10 with distilled water. All samples and positive and negative controls were prediluted (1/400) using the wash solution. Following the predilution, 100 μl of the positive and negative controls and samples were pipetted into antigen-coated wells. The plates were then tightly sealed and incubated at 37°C for 1 h. The plates were washed three times with the wash solution, and 100 μl of the conjugate was added to each well and incubated for 1 h at 37°C. Subsequently, 100 μl of TMB substrate N.12 was added to each well after washing three times with the wash solution. The plates were then incubated at 26°C for 15 min. After adding 100 μl of the stop solution N.3 into each well, the wavelength was read at 450 nm using a Thermo Lab systems Multiskan MS Original microplate reader (Thermo Fischer Scientific, Waltham, MA, United States). To validate the assay, the following procedures were used: The average optical density value of the two negative controls (NCx) at 450 nanometers (A450) must be equal to and not (≤) 0.500 for the assay to be valid. At 450 nanometers (A450), the two positive controls’ (PCx) average value should be less than or equal to (≤) 2.500. PCx-NCx (A450) must thus be greater than or equal to (≥) 0.300. The sample-to-positive control (S/P) ratio was calculated according to the formula:

$$S/P\%=100\times \frac{\mathrm{Sample}\left(A450\right)-\mathrm{NCx}\left(A450\right)}{\mathrm{PCx}\left(A450\right)-\mathrm{NCx}\left(A450\right)}$$

In terms of results interpretation, S/P % < 30% denoted a negative result, 30% ≤ S/P% < 40% denoted a suspect, while S/P% ≥40% denoted a positive result.

### 2.7. Molecular detection

#### 2.7.1. DNA extraction and PCR for detection of *Coxiella burnetii*

For extraction of DNA from sheath scrapings and vaginal swabs, the High Pure PCR template preparation kit (Roche Diagnostics GmbH, Mannheim, Germany) was used according to the manufacturer’s instructions. A sample volume of 1 ml from sheath scrapings/vaginal swabs in PBS was transferred to a 2 ml centrifuge tube and centrifuged at 1,467*g* for 10 min. A volume of the supernatant was discarded, leaving ~200 μl to re-suspend the pellet. Briefly, 40 μl of proteinase K and 200 μl of the binding buffer were added to 200 μl of the sample. Following incubation at 70°C for 10 min, 100 μl of isopropanol was added, and the mixture was then centrifuged at 8,000*g* for 1 min, and the flow-through and collection tube was discarded. A total volume of 500 μl of inhibitor removal buffer was added, the mixture was centrifuged at 8,000*g* for 1 min, and the flow-through and collection tube was discarded. This was followed by adding 500 μl of the wash buffer and centrifugation at 8,000*g* for 1 min and the flow-through and collection tube was discarded. Following the addition of 500 μl of wash buffer, the mixture was centrifuged at 8,000*g* for 1 min, and the flow-through was discarded. After centrifuging for 10 s at 13,000*g*, the collection tube was discarded, and a new tube was added, along with 200 μl of elution buffer, before centrifuging for 1 min at 8,000*g* and storing at −20°C. The extracted DNA was amplified using *Coxiella* IS*1111* PCR.

#### 2.7.2. Detection of *Coxiella burnetii* by PCR

The *Coxiella* IS*1111* PCR was conducted as previously described by Mangena et al. (10). The reactions for *C. burnetti* detection in seropositive and seronegative samples were performed in a 50 μl reaction targeting the multi-copy transposase gene in insertion element; IS*1111* (23) with primers IS*1111*F 5′CGCAGCACGTCAAACCG3′ and IS*1111*R 5′TATCTTTA ACAGCGCTTGAACGTC3′. The positive control used was an in-house DNA sample from Mangena et al. (10), which has been sequenced, and the negative control used was distilled water. The reaction mixture contained 2 μl of each 10 μM primer (IS*1111*F and IS*1111*R), 25 μl of the Amplicon 2X Taq DNA polymerase Master Mix Red (Amplicon A/S, Odense, Denmark), and 10 μl of the extracted DNA. For *C. burnetii* DNA amplification, a 146 bp sequence of the IS*1111* gene was amplified using BIO-RAD T100™ thermal cycler (BIO-RAD, Singapore) in which the first denaturation was at 95°C for 15 min, 35 cycles were carried out with denaturation at 95°C for the 30s, primer annealing for 30s at 60°C, followed by 72°C for 60 s and the final extension step at 72°C for 10 min.

#### 2.7.3. Gel electrophoresis

The amplicons from all the PCR assays were electrophoresed on a 1.5% ethidium bromide-stained agarose gel at 100 V for 1:30 h or until the separation of the DNA bands was observed. The size of the amplicons was determined using a 100 bp molecular weight marker. Gels were visualized under ultraviolet light using a gel documentation system (Chemi XRQ, Vacutec, United Kingdom).

### 2.8. Sequence verification

The PCR products were sent to Inqaba Biotechnologies, South Africa, for sequencing of both forward and reverse strands of the IS*1111* gene using an ABI sequencer. Due to financial constraints, only seven amplicons were sequenced. Sequences were edited manually, and pairwise alignments were undertaken using the BioEdit Sequence alignment editor (version 7.1.9) and the sequences were analyzed on the NCBI BLAST platform for species identification^2^ by megablast.

### 2.9. Data analysis

A univariable analysis using Chi-square test was applied to screen the variables, district municipality (Capricorn, Sekhukhune, Waterberg), type of farm (commercial, communal), herd size (1–50, 51–100, 101–150, >150), sex (female or male) and tick infestation or presence of rodents in the farms (yes or no), abortion history (yes or no), infertility history (yes or no), weak offspring history (yes or no) and manure uses (fertilizer or not used) against disease exposure at the individual level. In the initial analysis, all the variables were tested individually for their unconditional association with outcome using the Chi-square test (*p* ≤ 0.30). The variable that generated the highest *p >* 0.3 during univariate logistic regression analysis was excluded. Variables with a *p* ≤ 0.3 in the univariate analysis were used to develop a multilevel logistic regression model for each exposure. The logistic model was reduced by stepwise elimination removing variables with *p* > 0.05. Farm type and manure use were excluded from the ELISA multilevel logistic regression model analysis, and tick infestation or presence of rodents in the farms was excluded from the PCR multilevel logistic regression model analysis because they were confounding factors. This process was repeated until the model with the lowest Akaike’s second-order information criterion (AIC) was identified. The odds ratios with 95% confidence interval results were noted. Cohen’s Kappa agreement test between the PCR assay and ELISA was done. Data was captured and cleaned in a Microsoft Excel spreadsheet. All analysis was done using StataCorp. 2015. Stata Statistical Software: Release 14.2 College Station, TX: StataCorp LP.

## 3. Results

### 3.1. Seroprevalence and associated risk factors

In the univariable analyses, there were significant differences in seropositivity to *C. burnetii* antibodies between farms that use manure and those that do not use (*p* = 0.03). There were also significant associations between Q fever seroprevalence in cattle and herd size (*p* < 0.01), and farm type (communal and commercial; *p* = 0.02). There were no significant differences in seropositivity to *C. burnetii* antibodies in infertility history (*p* = 0.13) and abortion history (*p* = 0.20; Table 1). The final multivariable logistic regression model showed that for herd size, the odds of ELISA positivity were higher in farms that had a herd size of >150 (OR: 9.88; 95%CI: 3.92–24.89; *p <* 0.01) as compared to those that had cattle <50. Of the 383 cattle tested for antibodies against *C. burnetii*, the overall seroprevalence was 24.28% (93/383) (Table 2).

**Table 1:** Seroprevalence of *Coxiella burnetii* (Q fever) in cattle on farms in Limpopo province, South Africa, and univariable analysis of possible risk variables in cattle for Q fever seropositivity.

Risk factor	Number of animals sampled	Number testing positive	% seropositive	Chi-square *p*-value
District municipality				
Capricorn	127	27	21.26	0.35
Sekhukhune	123	28	22.76	
Waterberg	133	38	28.57	
Sex				
Female	335	81	24.18	0.90
Male	48	12	25.00	
Abortion history^a^				
No	158	33	20.89	0.20
Yes	225	60	26.67	
Infertility history^a^				
No	363	91	25.07	0.13
Yes	20	2	10.00	
Weak offspring history				
No	335	81	24.18	0.90
Yes	48	12	25.00	
Manure uses^a^				
Fertilizer	252	70	27.78	0.03
Not used	131	23	17.56	
Ticks infestation or presence of ticks in the farms				
No	54	14	25.93	0.76
Yes	329	79	24.01	
Herd size^a^				
1–50	191	36	18.85	<0.01
51–100	53	11	20.75	
101–150	112	27	24.11	
>150	27	19	70.37	
Farm type^a^				
Commercial	215	62	28.84	0.02
Communal	168	31	18.45	

a Variable significant (*p* < 0.30) for inclusion into multiple logistic regression model.

**Table 2:** Multivariable analysis of risk factors associated with seropositivity to *C. burnetii* in cattle farms in Limpopo province, South Africa.

Risk factor	OR	95% CI	Value of *p*
Abortion history			
Yes^a^			
No	1.09	0.64–1.87	0.74
Herd size			
1–50^a^			
51–100	1.48	0.64–3.44	0.36
101–150	1.38	0.78–2.46	0.26
>150	9.88	3.92–24.89	<0.01

Likelihood ratio Chi-squared = 2.31. Chi-squared probability = 0.13. OR, Odds ratio; CI, Confidence interval.

a Reference category.

### 3.2. Molecular prevalence and associated risk factors

**Figure 2:**
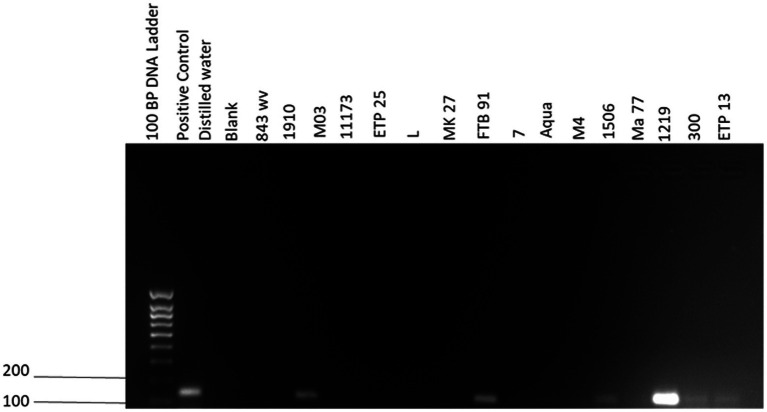
IS*1111* gel electrophoresis of PCR amplicons. Quick-Load^®^ 100 bp DNA ladder was used in the first lane (New England Biolabs, Ipswich, MA, United States). The positive control is an in-house positive control from Mangena et al. (10), the negative control is distilled water, and the blank is an empty lane. 843 wv, 1910, M03, 11173, ETP 25, L, MK27, FTB 91, 7, Aqua, M4, 1506, Ma 77, 1219, 300 and ETP 13 are cattle vaginal swab samples.

In the univariable analyses, we observed significant differences between PCR positivity and abortion history (*p* < 0.01) and herd size (*p* < 0.01). There were no significant differences in PCR positivity to tick infestation/presence of ticks (*p* = 0.07), manure uses (*p =* 0.18), sex (*p =* 0.14) and farm type (*p =* 0.13; Table 3). The odds of PCR positivity were higher in farms that had no abortion history compared to those that had abortion history. We also observed that the odds of PCR positivity were higher in farms with cattle >150 than farms with cattle <50 and between 50 and 150 (Table 4). The farms with no abortion history (OR: 0.37; 95%CI: 0.18–0.77; *p* < 0.01) and herd size of >150 (OR: 3.52; 95%CI: 1.34–9.24; *p* < 0.01) remained associated with *C. burnetii* positivity by PCR. Of the 383 cattle samples tested by IS*1111* PCR, 60 (15.67%) cattle were positive by PCR. Molecular detection of *C. burnetii* from sheath scrapings and vaginal swabs by PCR targeting the 262 IS1111 is illustrated as an example in Figure 2.

**Table 3:** Molecular prevalence of Q fever and univariable analysis of risk factors associated with PCR positivity to *C. burnetii* in cattle on farms in Limpopo province, South Africa.

Risk factor	Number of animals sampled	Number testing positive	% PCR positive	Chi-square *p*-value
Sex^a^				
Female	335	56	16.72	0.14
Male	48	4	8.33	
Abortion history^a^				
No	158	14	8.86	<0.01
Yes	225	46	20.44	
Manure uses^a^				
Fertilizer	252	44	17.46	0.18
Not used	131	16	12.21	
Ticks infestation or presence of ticks in the farms^a^				
No	54	13	24.07	0.07
Yes	329	47	14.29	
Herd size^a^				
1–50	191	24	12.57	<0.01
51–100	53	10	18.87	
101–150	112	14	12.50	
>150	27	12	44.44	
Farm type^a^				
Commercial	215	39	18.14	0.13
Communal	168	21	12.50	

a Variable significant (*p* < 0.30) for inclusion into multiple logistic regression model.

**Table 4:** Multivariable analysis of risk factors associated with PCR positivity to *C. burnetii* in cattle farms in Limpopo province, South Africa.

Risk factor	OR	95% CI	Value of *p*
Abortion history			
Yes^a^			
No	0.37	0.18–0.77	<0.01
Manure uses			
Fertilizer^a^			
Not used	0.73	0.36–1.46	0.37
Herd size			
1–50^a^			
51–100	1.84	0.72–4.72	0.20
101–150	1.10	0.53–2.28	0.79
>150	3.52	1.34–9.24	<0.01

Likelihood ratio Chi-squared = 2.31. Chi-squared probability = 0.13. OR, Odds ratio; CI, confidence interval.

a Reference category.

The Cohen’s kappa agreement test was done to assess if there is agreement between the PCR and ELISA results which revealed a kappa value of 0.40. Relatively fewer cattle, (60, 15.67%) were positive by PCR than by ELISA (93, 24.28%). Of the total ELISA-negative samples, (22, 7.59%) were positive by PCR and we observed a correlation of 93.41%. Of the overall ELISA-positive samples, there was an agreement of (38, 40.86%) positive by PCR. Out of all the 323 samples that were negative by PCR, (55, 20.52%) were positive by ELISA and a correlation of 79.48% was observed. Of the overall PCR-positive samples there was an agreement of (38, 63.33%) positive by ELISA assay.

## 4. Discussion and conclusions

A seroprevalence of 24.28% observed for Q fever in this study using indirect ELISA is the first evidence of *Coxiella burnetii* presence in cattle on farms in Limpopo province, South Africa. The seroprevalence observed in this study differs from other surveys conducted in the country. A survey conducted in the abattoirs in Gauteng province of South Africa by Mangena et al. (10) reported a lower seroprevalence of 9.40% than in the current study in cattle using an indirect ELISA. The seroprevalence noted in this study is lower than that in a survey by Adesiyun et al. (20), which reported a significantly higher prevalence of 38.9% in cattle in Bushbuckridge municipality, Mpumalanga province of South Africa, using an indirect ELISA. Gummow et al. (19) reported a seroprevalence of 7.8% using CFT in the Transvaal (Gauteng) province of South Africa, which is much lower than what was obtained in this study. The differences in the seroprevalence obtained in all these studies can be ascribed to factors such as study locations (Limpopo, Mpumalanga, Gauteng provinces of South Africa) and the years in which samples were collected (November 2020 to September 2021, April to September 2013, March 1985 to July 1986, respectively). These variables potentially influence exposure and detection of *C. burnetii* infections (24–26). The difference in the seroprevalence noted in all these studies may also be due to geographical variability. Previous studies have shown that, geographic region may influence seroprevalence (27–29). Mpumalanga has a subtropical climate with mild to cool winters that transition to cold and frosty conditions in the Highveld and an average temperature of 21°C which favors the development of pests such as ticks (30, 31). In Gauteng province, only the months of July and August experience the coldest weather, with an average daily temperature of 24°C. Limpopo has an average daily high temperature of 26°C. The majority of Limpopo province is warm all year, with little cold experienced during winter (30).

In comparison to other countries in Africa, the seroprevalence reported in the current study is comparable and falls within the range noted in other African countries. A study in Egypt revealed a seroprevalence of 19.3% in cattle using an indirect ELISA (32), which is slightly lower than the findings in the current study. Seroprevalences of 6.5% using complement fixation test and 32% using commercial ELISA kit were reported in Malawi and Cameroon’s Adamawa region cattle by Staley et al. (33) and Scolamacchia et al. (31), respectively. Mwololo et al. (13) reported a seroprevalence of 3.00% in cattle in Kenya’s Tana River and Garissa counties using iELISA.

Since we only sampled cattle in our study, we could not determine prevalence variation by animal species, and we also did not document any cattle housing system; however, the seroprevalence data inthe current study shows that the cattle in the study area are exposed to *Coxiella burnetii* with prevalence comparable to other studies (34). However, other studies, have shown that prevalence varies according to animal species (35, 36).

Farms with herd size >150 (*p* < 0.01) had a significantly higher risk of being seropositive to *C.burnetii* infection compared to farms with herd size between 1 and 50. The findings agree with the report of Cadmus et al. (15), who reported a higher seroprevalence in the herd size category of above 50 but a lower seroprevalence in smaller herds (10–30). Adamu et al. (37) demonstrated that the larger the herd size, the greater the risk of disease transmission through interaction with other infected animals or herds while grazing on contaminated pasture or sharing water stations.

This is the first study in South Africa to determine the molecular prevalence of *C.burnetii* in cattle. Other studies have reported molecular prevalence somewhere else. In the current study, we detected a lower molecular prevalence (15.67%) by PCR. Studies by Cardinale et al. (38) and Knobel et al. (6) reported lower molecular prevalences of 0.81 and 2.1% in cattle vaginal swabs samples in Reunion Island and western Kenya, respectively. A study conducted in India also reported a lower molecular prevalence of 1.81% in cattle vaginal swabs samples (39). Anastácio et al. (40) reported a higher prevalence (20.0%) in bulk tank milk samples in Portugal. This can be attributed to the type of sampling, the detection method used, and the types of samples since these could affect detection rates (10, 40, 41).

This current study demonstrated a significant association (*p* < 0.01) between *C. burnetii* positivity by PCR and cattle with no abortion history on the farms compared to those with history of abortion and this can be explained, in part, by the fact that infected ruminants can either be asymptomatic or symptomatic showing symptoms such as metritis, and mastitis (3). To reduce Q fever infection in cattle, vaccination with a phase 1 vaccine has been shown to reduce the occurrence of abortions and other reproductive problems in livestock (42, 43). However, South Africa has no policy plan for a cattle vaccination program.

The association between herd size and an increased risk of *C. burnetii* infection has been well-established (44, 45). In our study, we observed a high frequency (44.44%) of cattle shedding *C. burnetii* in the herd size category of >150. Also, we also discovered that farms with more than 150 cattle were more likely to be PCR positive (*p* < 0.01). This association can be explained, in part, by a larger population at risk, an increased risk of pathogen introduction and transmission within and between herds, such as increased feed and animal supply, and more professionals working at or visiting the farm (46). Larger herds are more likely to contract and develop Q fever, and the number of animals must be considered a risk factor for *C. burnetii* spread (40).

A comparison of the positivity detected by ELISA and PCR assay revealed a kappa value of 0.40 which is defined as a fair agreement by McHugh (47). In the current study, we observed a lower molecular prevalence detected by PCR compared to the seroprevalence of Q fever assayed by iELISA. This finding could be attributed to some of the cattle shedding *C. burnetii* through other routes (faecal or milk) (48), which were not assayed by PCR in this current study. These data also suggest that *C. burnetii* is widespread within the sampled areas. In this current study, ELISA positivity and PCR negativity indicated a past *C. burnetii* infection with no bacterial shedding through vaginal mucus in cows or preputial discharge in bulls. Infected animals shed *C. burnetii* in their faeces, vaginal discharges, and milk for several days or months after parturition (49–52). In contrast, the presence of PCR-positive samples and ELISA-negative samples could be explained by a recent *C. burnetii* infection in cattle in which antibodies have not yet been produced against the bacteria, or the level of these antibodies is below the detectability threshold (14, 40, 53). The presence of the pathogen in cattle and the evidence of exposure, as shown by both PCR and ELISA positivity in the current study suggests an active circulation of the pathogen, and possibly past exposure reflected by the ELISA results, as earlier reported (54).

## 5. Conclusion

Finally, the study examined the seroprevalence, PCR prevalence, and risk factors for *C. burnetii* in cattle on farms in South Africa’s Limpopo province. This study determined that *C. burnetii* is present in the study areas (Capricorn, Waterberg, and Sekhukhune). Herd size with cattle >150, was significantly associated with Q fever seroprevalence. Cattle or farms with no abortion history and herd size with cattle >150 were found to be significantly associated with Q fever molecular prevalence as detected by PCR. *Coxiella burnetii* should be considered a possible source of human Q fever in the study areas and widespread in cattle.

## 6. Limitations

The present study had limitations, including the number of samples collected lower than the calculated sample size for the survey. Failure to sample two local municipalities due to financial constraints, resulted in a smaller sample size and two districts municipalities that are FMD-restricted areas. Some farmers in the sampled district municipalities were unwilling to participate in the study and were thus excluded.

## 7. Recommendations

Conduct large-scale studies in South Africa in animals and people to determine the prevalence of *C. burnetii* and the risk factors of *C. burnetii* infection in humans. Management measures should be developed based on the identified risk factors for exposure to Q fever. Future studies should investigate the seroprevalence and PCR prevalence of *C. burnetii* infection to differentiate between past and current exposure and conduct a molecular characterization of the pathogen to determine the strains circulating in the country. Other studies should be conducted on ticks and *Coxiella*-like bacteria. A long-term plan for animal vaccination should be developed to reduce the number of infections and animal-to-human transmission. Awareness campaigns should also be launched to educate farmers about the risks of Q fever and proper hygiene practices. It is recommended that a surveillance system for Q fever control and prevention be developed in South Africa.

## Data availability statement

The original contributions presented in the study are included in the article/supplementary material, further inquiries can be directed to the corresponding author.

## Ethics statement

The animal study was reviewed and approved by Faculty of Veterinary Science (University of Pretoria).

## Author contributions

VS conducted the experiments, wrote, and edited the manuscript. NG, MM, and AA equally conceptualized, wrote, edited, and reviewed the manuscript while YN conducted statistical analysis of the data and co-authored the manuscript. All authors contributed to the article and approved the submitted version.

## Funding

Financial support to conduct the study was obtained from the Red Meat Research and Development in South Africa (RMRD-SA) and the Department of Trade, Industry, and Competition (DTIC), South Africa – THRIP (THRIP/23/30/11/2017).

## Conflict of interest

The authors declare that the research was conducted in the absence of any commercial or financial relationships that could be construed as a potential conflict of interest.

## Publisher’s note

All claims expressed in this article are solely those of the authors and do not necessarily represent those of their affiliated organizations, or those of the publisher, the editors and the reviewers. Any product that may be evaluated in this article, or claim that may be made by its manufacturer, is not guaranteed or endorsed by the publisher.

## Footnotes

^1^ https://municipalities.co.za

^2^ https://blast.ncbi.nlm.nih.gov/Blast.cgi
